# Lack of Associations between Environmental Exposures and Environmental Enteric Dysfunction among 18-Month-Old Children in Rural Malawi

**DOI:** 10.3390/ijerph191710891

**Published:** 2022-09-01

**Authors:** Zhifei Liu, Yue-Mei Fan, Per Ashorn, Chilungamo Chingwanda, Kenneth Maleta, Lotta Hallamaa, Heikki Hyöty, David Chaima, Ulla Ashorn

**Affiliations:** 1 Center for Child, Adolescent and Maternal Health Research, Faculty of Medicine and Health Technology, Tampere University, 33014 Tampere, Finland; 2 Department of Paediatrics, Tampere University Hospital, 33521 Tampere, Finland; 3 School of Public Health & Family Medicine, Kamuzu University of Health Sciences, Private Bag 360, Chichiri, Blantyre 3, Malawi; 4 Fimlab Laboratories, Pirkanmaa Hospital District, 33521 Tampere, Finland

**Keywords:** environmental exposure, calprotectin, alpha-1-antitrypsin, REG1B, Malawi

## Abstract

Environmental enteric dysfunction (EED) is common and contributes to linear growth faltering (stunting) and mortality among children in low-resource settings. A few studies on the environmental causes of EED have been conducted but the exact exposures that cause or predispose children to EED are context-specific and not clear. This study aimed to assess associations between selected environmental exposures and EED markers among 620 18-month-old children. This was a secondary analysis of data from Malawian children who participated in a randomized controlled trial (iLiNS-DYAD, registered at clinicaltrials.gov as NCT01239693) from birth to 18 months of age. Data on environmental exposures, including drinking water source, sanitation, exposure to animals, housing materials, season, residential area, and food insecurity were collected at enrolment. Biomarkers of EED included concentrations of calprotectin, regenerating 1B protein (REG1B), and alpha-1-antitrypsin from stool samples to assess intestinal inflammation, repair, and permeability, respectively. We performed bivariate and multivariable analyses to assess associations between environmental exposures and EED biomarkers. Adjusting for possible confounders, we did not find associations between the selected environmental exposures and the three biomarkers. These results do not provide support for our hypothesis that the studied adverse environmental exposures are associated with increased concentrations of children’s EED markers in rural Malawi.

## 1. Introduction

Environmental enteric dysfunction (EED) is a common condition among children in low-income settings. It is characterized by increased intestinal permeability, intestinal inflammation, and impaired barrier function [1,2]. EED typically starts in infancy and continues into adulthood [1,3]. Growing evidence has demonstrated that EED has a strong association with stunting which contributes to more than one million deaths among children under 5 years old every year [4,5].

As the name implies, EED is considered environmentally caused [1,3], but the exact factors in the environment resulting in this condition are not well understood. Typically, these factors include unsafe drinking water, poor sanitary facilities, exposure to animals, and season [1]. Several studies have demonstrated that unsafe drinking water and sanitation, exposure to chickens, cows, and goats, and the rainy season may be risk factors for EED [6,7,8,9,10,11]. However, an intervention addressing water, sanitation, and hygiene (WASH) do not have a positive impact on reducing EED in rural Zimbabwe [12]. Consequently, there are inconsistent data about the association between environmental factors and EED. Additionally, other factors including geographical context, food insecurity, and housing materials contribute to stunting, which is strongly associated with EED, but their associations with EED are poorly understood [5,13,14,15,16,17]. Evidence that these potential environmental factors are associated with EED concomitantly is lacking especially in young children.

To assess the associations of EED with environmental factors concomitantly, we considered other factors as covariates which have been associated with intestinal health [18,19]. In particular, this included breastfeeding, dietary supplementation with small-quantity lipid-based nutrient supplements (SQ-LNS) and antibiotic use, which were reported to be associated with gut microbiome [20,21,22]. Our study aimed to investigate whether there would be associations between these exposures and EED in 18-month-old rural Malawian children. As biomarkers of EED, we used fecal concentrations of calprotectin, regenerating 1B protein (REG1B), and alpha-1-antitrypsin, indicating inflammation, cellular repair, and permeability in the gastrointestinal tract [23,24,25,26]. We hypothesized that exposure to adverse environmental conditions would be associated with increased fecal concentrations of these three biomarkers among rural Malawian children.

## 2. Materials and Methods

### 2.1. Study Participants

The present study was a secondary analysis of data from a randomized controlled trial (iLiNS-DYAD trial) on the effects of lipid-based nutrient supplements on pregnant women and infants in Mangochi district, Malawi [27]. In brief, pregnant women with less than 20 completed weeks gestation were enrolled and randomized to three different groups in which they received different nutrient supplements: iron and folic acid (IFA), multiple micronutrients (MMN), or SQ-LNS. The current analysis included newborns who were followed-up until 18 months of age. Infants whose mothers had received SQ-LNS during pregnancy received 20 g/day SQ-LNS from 6 to 18 months and other infants received no supplements. The iLiNS-DYAD trial was prospectively registered at clinicaltrials.gov as NCT01239693.

Main efficacy analyses from the trial data do not support a hypothesis that provision of SQ-LNS to pregnant women and their children would increase the infants’ birth weight or growth by 18 months of age [27,28]. In the current ancillary study to the trial, we analyzed the associations between selected environmental exposures and intestinal health among the participating children at 18 months of age.

Before enrolment, all pregnant women gave a signed informed consent for participation and another consent was given before enrolment of their children. The original trial was approved by the ethics committees of College of Medicine in Malawi and Pirkanmaa Hospital District in Finland.

### 2.2. Environmental Exposures

We selected the following environmental exposures for this analysis based on our previous study [19]: drinking water source (well, lake and river vs. piped water and borehole); sanitation (regular pit latrine and none vs. water closet and ventilated improved pit latrine); exposure to chickens, goats or cows, representing exposure to feces of these animals possibly causing intestinal infection [29,30]; wall material of the main house (unburnt brick, straw, grass or mud vs. burnt brick); roofing material of the main house (grass and other vs. iron sheets or tiles); season when children’s stool samples were collected (rainy in December–March and hot-dry in August–November vs. cold-dry in April–July); residential area (Malindi, Mangochi, and Lungwena); and food insecurity score (food secure, mildly food insecure, moderately food insecure, and severely food insecure), indicating if food intake or eating patterns were disrupted due to physical or economic conditions in the household in the past month [31,32].

Research assistants collected information on the environmental exposures using questionnaires when mothers were enrolled. In addition, the data on exposure to animals were recorded using questionnaires a few days before the collection of children’s stool samples at 18 months of age.

### 2.3. Outcomes

We included concentrations of calprotectin, REG1B, and alpha-1-antitrypsin in stools at 18 months as intestinal health outcomes in this analysis. These biomarkers of EED have been validated for measuring intestinal health in pediatric populations [12,23,33]. Increase in concentrations of these biomarkers reflected increased inflammation, repair, or permeability in gastrointestinal tract, respectively [24,34].

Research assistants collected stool samples from children during home visits and stored the samples in cryovial tubes at −20 °C in local laboratories. Within 48 h, these samples were transported to a central laboratory and stored at −80 °C before they were shipped to Tampere University in Finland for laboratory analysis. If the child had diarrhea at home visit, the stool collection from these children was postponed for 2 weeks. To measure fecal concentrations of these three biomarkers, we used commercial enzyme-linked immunosorbent assay (ELISA) kits for calprotectin (Hycult Biotech, Uden, The Netherlands), REG1B (TECHLAB, Blacksburg, VA, USA), and alpha-1-antitrypsin (PromoCell GmbH, Heidelberg, Germany). We chose these kits based on their suitability and sensitivity [34,35,36]. These kits provided lower limit of detection (LOD) which was 16 µg/g for calprotectin, 6.25 µg/g for REG1B, and 1.8 mg/dL for alpha-1-antitrypsin. Briefly, we thawed and weighed around 100 mg of stools for calprotectin or alpha-1-antitrypsin, and around 50 mg for REG1B. A dilution factor of 1:50 was used for calprotectin, 1:10,000 for REG1B and 1:250 for alpha-1-antitrypsin. The final concentration was calculated based on the respective standard curve plotted with the absorbance versus different concentrations of standards.

### 2.4. Other Variables

Based on previous research, we considered the following variables as possible covariates in our analyses [19,20,21,22,37,38]: maternal age, maternal HIV infection status (infected/not infected), maternal educational achievement in years, child sex, weight-for-length z-score (WLZ) and length-for-age z-score (LAZ) at 18 months, continued breastfeeding (yes/no), and number of days of antibiotic use between birth and 18 months of age (above/below median days) in children.

We obtained information on maternal age and educational achievement using questionnaires at enrollment and data on HIV infection status using a whole-blood antibody rapid diagnosis test (Alere Determine™ HIV-1/2, Abbott Alere, Matsudo, Japan). We collected data on child sex from health records at birth, and data on continued breastfeeding by questionnaires at 18 months. Length and weight at 18 months were assessed using a length board (Harpenden Infantometer, Holtain Limited, Crosswell, UK) and an infant weighing scale (SECA 735). WLZ and LAZ were calculated based on World Health Organization (WHO) Child Growth Standards [39]. Data on antibiotic use were extracted from medical records from birth until 18 months.

### 2.5. Data Analysis

We performed data analyses using Stata software version 15.0 (StataCorp, College Station, TX, USA). We described continuous variables using mean (standard deviation) and categorical variables using percentage.

For the original values of EED biomarker concentrations lower than LOD, we did not repeat measurements due to limited amounts of stool samples. We replaced these values with a half of LOD, which was recommended to be used by WHO GEMS/Food-EURO workshop [40]. Due to skewed distribution of biomarker concentrations, we used log-transformed values as outcomes in the models. We used Pearson’s correlation coefficients to estimate the correlation between these EED biomarkers. The correlation was considered strong with coefficient >0.5; moderate with coefficient >0.3 but <0.5; and weak with coefficient >0.1 but <0.3 [41]. We assessed the association between environmental exposures and EED biomarkers by bivariate analysis and multivariable analysis using linear regression. For multivariable analysis, our models were adjusted for all environmental exposures and the following background characteristics: maternal age, HIV infection status, educational achievement, child sex, WLZ and LAZ at 18 months, continued breastfeeding, antibiotic use, and dietary intervention. For hypothesis testing, a *p*-value < 0.05 was deemed statistically significant. Considering possible effect medication by the child’s sex, we included sex *environmental exposures variables in multivariable models for EED biomarkers.

We checked multicollinearity by a variance inflation factor (VIF) tool and removed environmental exposures from multivariable models if they contributed to multicollinearity. The exclusion criteria included a VIF-value of more than 1 and at the same time a change in any *p*-value from statistically significant to non-significant when the respective exposure was removed from the multivariable model. To assess the robustness of our multivariable models, we performed a sensitivity analysis in which missing data in independent variables were imputed by chained equations with 20 imputed datasets.

## 3. Results

**Figure 1:**
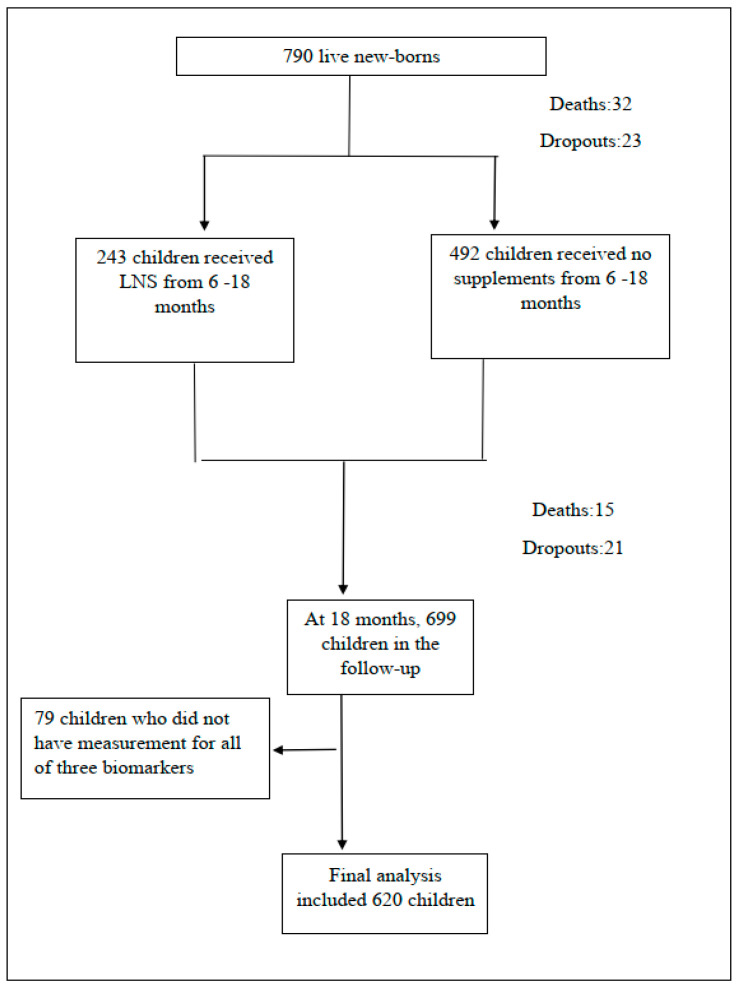
Flow chart of participants.

Of the 790 live-born infants enrolled in the study, 47 (6%) died and 44 (6%) dropped out from the follow-up by 18 months of age. Another 79 children were excluded because of missing biomarker data, leaving data from 620 children in the analysis (Figure 1).

There were no differences in anthropometric indicators at 18 months of age between the included and excluded children, but children whose data were excluded from the analysis had mothers with higher mean educational achievement (5 years vs. 4 years) and a lower proportion of continued breastfeeding than those included in the analysis (83% vs. 93%). The participants’ mean WLZ and LAZ at 18 months were −0.2 and −1.7, respectively; the prevalence of wasting and stunting was 4% and 37%, respectively (Table 1).

**Figure 2:**
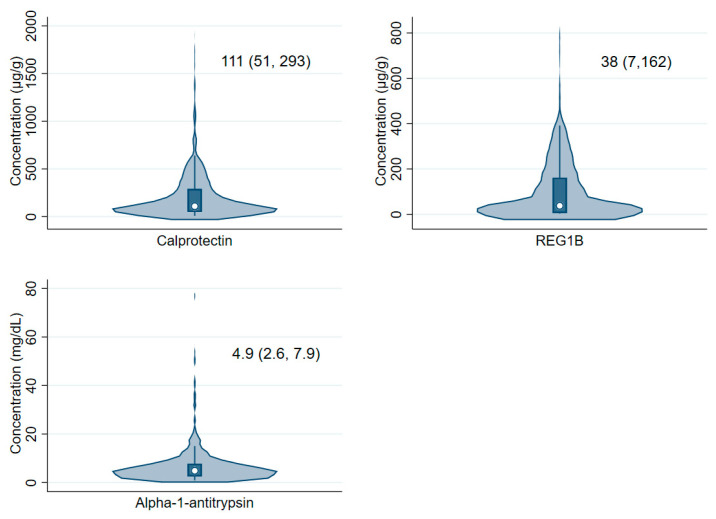
The distribution of concentrations of fecal calprotectin, REG1B, and alpha-1-antitrypsin at 18 months of age.

The median (interquartile range) concentrations of calprotectin, REG1B, and alpha-1-antitrypsin at 18 months were 111 µg/g (51, 293), 38 µg/g (7, 162), and 4.9 mg/dL (2.6, 7.9), respectively (Figure 2). There was a strong positive correlation between calprotectin and alpha-1-antitryspsin level, and weak positive correlations between calprotectin and REG1B and between REG1B and alpha-1-antitrypsin (Table S1).

In the bivariate analysis, most of the selected environmental exposures were not associated with concentrations of EED biomarkers (Table 2). As an exception, there was an association between the season of stool sample collection and the stool’s calprotectin and alpha-1-antitrypsin concentration and between the type of sanitary facility in the participants’ home and calprotectin concentration. The children’s stool biomarker concentrations were on average higher in rainy season or hot-dry season than in cold-dry season and lower in households with no sanitary facility or a regular pit latrine than in households with a water closet or a ventilated improved pit latrine (Table 2).

Roofing material used as one of environmental exposures, was excluded from the multivariable analysis due to multicollinearity (Table S2). The analyses of adjustment for covariates did not change the association between the season of stool sample collection and the stool’s calprotectin concentration but removed that between the season and alpha-1-antitrypsin concentration (Table 3). The adjusted analyses also indicated an association between the participants’ residential area and REG1B concentration, with participants living in Mangochi having a lower concentration on average than those in Lungwena (Table 3).

Results from the sensitivity analysis on the association between EED biomarkers and environmental exposures with imputed data were consistent with those in the multivariable analysis (Table S3).

For covariates earlier associated with gut microbiome, increased antibiotic use was associated with lower concentrations of calprotectin (*p* = 0.002) and alpha-1-antitrypsin (*p* = 0.009). There were no associations between continued breastfeeding or dietary supplementation with SQ-LNS and any of these biomarkers (*p* > 0.05, Table S4).

Inclusion of sex *environmental exposures variables in multivariable models for EED biomarkers did not show any indication of sex differences in associations between the studied environmental exposures and EED biomarkers.

## 4. Discussion

Our analysis explored the association between multiple environmental exposures and EED. In a sample of 620 rural Malawian 18-month-old children, after adjusting for potential confounders, we found no associations between most of the selected environmental exposures and fecal concentrations of calprotectin, REG1B, and alpha-1-antitrypsin which represented intestinal inflammation, repair, and permeability, respectively. We only observed two associations between the season and calprotectin concentration and between residential area and REG1B concentration.

Even though these two associations were found in our samples, they were less likely to be indications of similar associations between these two environmental exposures and EED in our target population which our samples were drawn from. This may be explained by the correlations between EED biomarkers and the consistency between different biomarkers. Our three biomarkers were correlated with each other, which has been indicated by a previous study in Bangladesh [34]. However, the season and the residential area were not consistently associated with the other two markers. Moreover, considering the complexity of the pathways of EED [42], it is likely that the consistency of associations between different biomarkers and the same environmental exposure is needed, but we did not see the associations between the season or the residential area and other two biomarkers. Therefore, it is more likely that this sample finding of associations between the season or the residential area and one single EED biomarker is spurious.

There are several factors related to the study design and implementation that could theoretically introduce biases in the results. These factors include number of excluded children, selection of covariates, utility of EED biomarkers, missing data, and reduction in sample size due to the selection of analysis models. However, the excluded children had on average similar characteristics compared with those included children. We included covariates that had been considered appropriate in previous studies such as breastfeeding and antibiotic use [20,21,43,44,45,46,47,48,49] to minimize the possible bias [50] in multivariable analysis. For biomarkers, calprotectin, REG1B, and alpha-1-antitrypsin have been widely used to study typical features of EED [34,51]. We also confirmed the robustness of our results using multiple imputations for missing data in the sensitivity analysis with multiple linear regression models [52]. Therefore, we believe that our sample findings are valid and representative of the target population.

The lack of association between EED and drinking water source, sanitation, or exposure to animals in rural Malawian children partially contradicts previous results from other study sites [6,7,8,9]. Similar to ours, the study in Peru provided no interventions to the living environment and used fecal alpha-1-antitrypsin as one of EED biomarkers. However, different features such as higher median concentration of alpha-1-antitrypsin at 18 months (0.35 mg/g vs. 0.049 mg/g), the use of myeloperoxidase and neopterin as markers of intestinal inflammation, multiple measurements for markers, and different classifications for exposures [9], might contribute to different findings from Peru. The MAL-ED study across multiple sites and two studies in Bangladesh measured environmental exposures using a joint index for drinking water source, sanitation facilities, and other socioeconomic factors or created an EED score by combining EED markers [6,7,8], while we considered each environmental exposure as a separate element and used each EED marker as the respective outcome. These differences in exposure and outcome definitions make it difficult to draw a more generic conclusion on the association between drinking water source, sanitation, exposure to animals and EED markers among children in low-income settings.

Elevated food insecurity in rural Malawi might contribute to worsening EED when there are insufficient food and drinking water in the dry season, according to the speculations from a study on the effects of zinc and albendazole treatments on EED [14]. In our sample, we did not see this association. Regarding housing materials, roofing material was excluded from the analysis due to multicollinearity and the adjusted model included only the wall material. Earlier studies have indicated a weak association with an intestinal problem (diarrhea) and housing materials [53], but we did not see an association of housing materials with EED in the Malawian context.

In our study, antibiotic use was inversely associated with calprotectin and alpha-1-antitrypsin concentrations in stools. This result is consistent with findings of a previous study from the iLiNS-DYAD trial, documenting an inverse relationship between antibiotic use and microbiota maturity and diversity that were associated with our three EED biomarkers [18,19].

Notably, improved latrine was associated with higher concentration of calprotectin compared to regular pit latrine and no latrine in bivariate analysis. A longitudinal study in Peru [9] provided similar results that improved sanitation facilities were associated with higher concentration of myeloperoxidase (a marker of intestinal inflammation). This may be explained by the location of latrines with unproper management of fecal sludge [9,54,55,56]. Higher level of intestinal inflammation may be due to more exposure to pathogens in soil surrounding the main house which was close to the improved latrine [56,57]. The proportion of households having improved latrine only accounted for 10% in our study. Therefore, it is possible that this proportion is too small to capture differences in concentrations of EED biomarkers between children living with different types of latrines in adjusted models.

## 5. Conclusions

Our results suggest that the selected environmental exposures are not associated with EED markers of children in the rural Malawian setting. These findings contribute to scientific evidence on the possible environmental causes of young children’s intestinal conditions such as EED in Sub-Saharan Africa.

## Figures and Tables

**Table 1:** Characteristics of participants included and excluded ^†^.

Characteristic	Included *N* = 620	Excluded *N* = 170	*p*-Value ^‡^
Maternal age, years	25 (6)	24 (6)	0.077
Mother, HIV infected ^a^	77 (12%)	17 (10%)	0.588
Educational achievement, years	4 (3)	5 (4)	<0.001 **
Child sex, boys	299 (48%)	73 (46%)	0.595
WLZ at 18 months	−0.2 (1.0)	−0.1 (0.9)	0.606
WLZ at 18 months < −2, wasting	22 (4%)	4 (0%)	0.250
LAZ at 18 months	−1.7 (1.1)	−1.6 (1.1)	0.600
LAZ at 18months < −2, stunting	226 (37%)	18 (29%)	0.267
Continued breastfeeding by 18 months	537 (93%)	40 (83%)	0.045 *
Number of days of antibiotic use between birth and 18 months	8 (18)	13 (20)	0.135

^†^ Values were mean (SD) or N(%). ^‡^ *p* value was obtained from student *t* test for continuous variables, and Fisher’s exact test for categorical variables. * *p* < 0.05, ** *p* < 0.01. ^a^ HIV, human immunodeficiency virus, LAZ, length-for-age z-score, WLZ, weight-for-length z-score.

**Table 2:** Associations between environmental exposures and intestinal biomarkers in bivariate analysis ^†^.

Environmental Exposures	Calprotectin		^§^ REG1B		Alpha-1-antitrypsin	
	Coef. (95%CI)	*p*-Value	Coef. (95%CI)	*p*-Value	Coef. (95%CI)	*p*-Value
Well, lake and river as drinking water source, 543/620 (vs. *piped water and borehole*)	−0.25 (−0.54, 0.05)	0.100	−0.03 (−0.48, 0.42)	0.899	0.03 (−0.20, 0.27)	0.785
Regular pit latrine and none, 563/620 (vs. *improved latrine*)	−0.32 (−0.64, −0.01)	0.045 *	−0.11 (−0.59, 0.38)	0.665	−0.16 (−0.42, 0.09)	0.205
Exposure to chickens, 303/620 (vs. *not exposure*)	−0.00 (−0.19, 0.18)	0.977	0.04 (−0.24, 0.32)	0.793	0.04 (−0.11, 0.19)	0.604
Exposure to goats, 147/620 (vs. *not exposure*)	−0.01 (−0.23, 0.20)	0.916	−0.20 (−0.53, 0.13)	0.242	0.00 (−0.17, 0.18)	0.974
Exposure to cows, 16/620 (vs. *not exposure*)	0.31 (−0.27, 0.88)	0.295	0.56 (−0.32, 1.45)	0.212	0.06 (−0.40, 0.52)	0.802
Poor quality wall of the main house, 391/620 (vs. *burnt brick*)	−0.19 (−0.38, 0.00)	0.051	0.11(−0.18, 0.40)	0.455	−0.14 (−0.29, 0.01)	0.069
Poor quality roofing material of the main house, 498/620 (vs. *iron sheets or tiles*)	−0.03 (−0.26, 0.20)	0.818	−0.08 (−0.44, 0.27)	0.649	−0.13 (−0.31, 0.06)	0.171
*Season* (vs. *Cold-dry*)						
Rainy, 216/620	0.45 (0.23, 0.67)	<0.001 **	−0.06 (−0.41, 0.29)	0.750	0.20 (0.01, 0.38)	0.034 *
Hot-dry, 218/620	0.43 (0.21, 0.66)	<0.001 **	−0.02 (−0.37, 0.33)	0.923	0.13 (−0.05, 0.31)	0.154
*Residential area* (vs. *Lungwena*)						
Malindi, 117/620	0.23 (−0.01, 0.47)	0.066	−0.05 (−0.43, 0.32)	0.789	0.10 (−0.09, 0.30)	0.297
Mangochi 171/620	0.09 (−0.12, 0.31)	0.386	−0.28 (−0.60, 0.05)	0.100	0.13 (−0.04, 0.30)	0.144
*Food insecurity* (vs. *food secure*)						
Mildly food insecure, 76/613	−0.09 (−0.44, 0.26)	0.618	0.16 (−0.38, 0.69)	0.562	−0.06 (−0.33, 0.22)	0.684
Moderately food insecure, 196/613	−0.07 (−0.36, 0.21)	0.606	0.05 (−0.38, 0.48)	0.813	−0.14 (−0.36, 0.09)	0.229
Severely food insecure, 243/613	−0.04 (−0.32, 0.23)	0.750	−0.18 (−0.60, 0.24)	0.403	−0.02 (−0.23, 0.20)	0.862

^†^ Results were from simple linear regression models. * *p*-value < 0.05. ** *p*-value < 0.01. ^§^ REG1B, regenerating 1B protein.

**Table 3:** Associations between environmental exposures and intestinal biomarkers in multivariable analysis ^†^.

Environmental Exposures	Calprotectin		^§^ REG1B		Alpha-1-antitrypsin	
	Coef. (95%CI)	*p*-Value	Coef. (95%CI)	*p*-Value	Coef. (95%CI)	*p*-Value
	*N* = 558		*N* = 558		*N* = 558	
Well, lake and river as drinking water source, 543/620 (vs. *piped water and borehole*)	−0.14 (−0.45, 0.17)	0.379	−0.06 (−0.54, 0.41)	0.793	0.10 (−0.15, 0.34)	0.431
Regular pit latrine and none, 563/620 (vs. *improved latrine*)	−0.22 (−0.56, 0.13)	0.216	−0.10 (−0.64, 0.43)	0.708	−0.05 (−0.32, 0.23)	0.730
Exposure to chicken, 303/620 (vs. *not exposure*)	−0.02 (−0.22, 0.18)	0.858	0.06 (−0.25, 0.37)	0.703	0.02 (−0.14, 0.18)	0.806
Exposure to goats, 147/620 (vs. *not exposure*)	−0.00 (−0.23, 0.23)	0.996	−0.22 (−0.58, 0.14)	0.228	0.02 (−0.17, 0.20)	0.847
Exposure to cows, 16/620 (vs. *not exposure*)	0.24 (−0.35, 0.82)	0.425	0.64 (−0.27, 1.54)	0.168	−0.03 (−0.49, 0.43)	0.904
Poor quality wall material of the main house, 391/620 (vs. *burnt brick*)	−0.10 (−0.31, 0.11)	0.367	0.22 (−0.11, 0.55)	0.195	−0.05 (−0.22, 0.11)	0.525
*Season* (vs. *Cold-dry*)						
Rainy, 216/620	0.39 (0.13, 0.65)	0.003 **	−0.07 (−0.47, 0.32)	0.715	0.16 (−0.05, 0.36)	0.133
Hot-dry, 218/620	0.37 (0.12, 0.62)	0.004 **	0.04 (−0.35, 0.43)	0.846	0.10 (−0.10, 0.30)	0.315
*Residential area* (vs. *Lungwena*)						
Malindi, 117/620	0.02 (−0.26, 0.31)	0.868	−0.10 (−0.54, 0.35)	0.670	0.04 (−0.18, 0.27)	0.698
Mangochi, 171/620	−0.08 (−0.34, 0.18)	0.547	−0.45 (−0.86, −0.04)	0.031 *	0.02 (−0.19, 0.23)	0.873
*Food insecurity* (vs. *food secure*)						
Mildly food insecure, 76/613	0.02 (−0.37, 0.42)	0.910	0.27 (−0.34, 0.88)	0.388	−0.01 (−0.32, 0.30)	0.940
Moderately food insecure, 196/613	−0.01 (−0.33, 0.32)	0.970	0.05 (−0.45, 0.56)	0.834	−0.08 (−0.34, 0.18)	0.548
Severely food insecure, 243/613	0.08 (−0.24, 0.40)	0.612	−0.13 (−0.63, 0.36)	0.595	0.10 (−0.15, 0.35)	0.430

^†^ Results were from multivariable regression models adjusted for other environmental exposures from bivariate analysis, maternal age, educational achievement, HIV infection status, child sex, WLZ and LAZ at 18 months, continued breastfeeding status, antibiotic use and dietary intervention. * *p*-value < 0.05, ** *p*-value < 0.01. ^§^ REG1B, regenerating 1B protein.

## Data Availability

The data will be available from the authors upon reasonable request.

## Supplementary Materials

The following supporting information can be downloaded at: https://www.mdpi.com/article/10.3390/ijerph191710891/s1, Table S1: Pearson’s correlations between biomarkers of intestinal health; Table S2: Values of VIF for environmental exposures; Table S3: Associations between environmental exposures and intestinal biomarkers in multivariable analysis after imputation; Table S4: Associations between breastfeeding, antibiotic use, and dietary intervention and intestinal biomarkers in multivariable analysis.
