# Supplementary material for: Lack of Associations between Environmental Exposures and Environmental Enteric Dysfunction among 18-Month-Old Children in Rural Malawi
Source: Int J Environ Res Public Health. 2022 Sep 1;19(17):10891. doi: 10.3390/ijerph191710891 (PMC9517768; doi:10.3390/ijerph191710891)
Supplement: Supplementary file 1 [file ijerph-19-10891-s001.zip › ijerph-1809950-SI.pdf]

Table S1. Pearson's correlations between biomarkers of intestinal health †.

	Calprotectin N=620		§ REG1B N=620		Alpha-1-antitrypsin N=620	
	**Coefficient**	***p*-Value**	**Coefficient**	***p*-Value**	**Coefficient**	***p*-Value**
Calprotectin	1	--	--	--	--	--
§ REG1B	0.19	<0.001**	1	--	--	--
Alpha-1-antitrypsin	0.57	<0.001**	0.20	<0.001**	1	--

† Results were from Pearson's correlation analysis. ** *p*-value < 0.01. § REG1B, regenerating 1B protein

Table S2. Values of VIF for environmental exposures †.

Environmental Exposure	VIF §	VIF when Excluding Variable "Roofing Material"
Drinking water source	1.02	1.02
Sanitary facility	1.14	1.11
Exposure to chickens	1.09	1.08
Exposure to goats	1.10	1.10
Exposure to cows	1.05	1.04
Wall material	1.52	1.12
Roofing material	1.71	-
Season	1.06	1.06
Residential area	1.30	1.29
Food insecurity	1.15	1.15

† VIF was calculated from multivariable regression models. § VIF, variance inflation factor.

Table S3. Associations between environmental exposures and intestinal biomarkers in multivariable analysis after imputation †.

Environmental Exposures	Calprotectin		§ REG1B		Alpha-1-antitrypsin	
	Coef. (95%CI)	*p*-Value	Coef. (95%CI)	*p*-Value	Coef. (95%CI)	*p*-Value
	N=620		N=620		N=620	
Well, lake and river as drinking water source (*vs. piped water and borehole*)	−0.17(−0.46,0.12)	0.255	−0.06(−0.52,0.39)	0.781	0.06(−0.17,0.30)	0.597
Regular pit latrine and none (*vs. improved latrine*)	−0.26(−0.59,0.07)	0.117	−0.14(−0.66,0.37)	0.585	−0.09(−0.35,0.18)	0.521
Exposure to chicken (*vs. not exposure*)	0.04(−0.15,0.22)	0.702	0.06(−0.24,0.35)	0.706	0.07(−0.08,0.22)	0.360
Exposure to goats (*vs. not exposure*)	−0.01(−0.24,0.21)	0.899	−0.20(−0.55,0.15)	0.258	−0.00(−0.18,0.18)	0.975
Exposure to cows (*vs. not exposure*)	0.23(−0.35,0.81)	0.431	0.61(−0.29,1.51)	0.186	−0.01(−0.47,0.46)	0.979
Poor quality wall material of the main house (*vs. burnt brick*)	−0.10(−0.30,0.11)	0.350	0.16(−0.16,0.47)	0.324	−0.07(−0.24,0.09)	0.386
Season (vs. Cold-dry)						
Rainy	0.39(0.15,0.63)	0.002**	−0.02(−0.40,0.36)	0.931	0.16(−0.04,0.35)	0.112
Hot-dry	0.40(0.17,0.63)	0.001**	0.03(−0.33,0.40)	0.855	0.10(−0.09,0.29)	0.285
Residential area (vs. Lungwena)						
Malindi	0.01(−0.26,0.29)	0.925	−0.21(−0.64,0.21)	0.326	−0.00(−0.22,0.22)	0.992
Mangochi	−0.10(−0.34,0.14)	0.426	−0.55(−0.93,−0.17)	0.005**	−0.01(−0.21,0.19)	0.927
Food insecurity (vs. food secure)						
Mildly food insecure	0.04(−0.31,0.40)	0.811	0.12(−0.44,0.67)	0.675	0.01(−0.28,0.30)	0.938
Moderately food insecure	0.03(−0.27,0.33)	0.856	0.05(−0.42,0.51)	0.847	−0.06(−0.30,0.19)	0.642
Severely food insecure	0.10(−0.19,0.40)	0.484	−0.14(−0.59,0.32)	0.551	0.08(−0.15,0.32)	0.486

† Results were from multivariable regression models adjusted for other environmental exposures from bivariate analysis, maternal age, educational achievement, HIV infection status, child sex, WLZ and LAZ at 18 months, continued breastfeeding status, antibiotic use and dietary intervention. ** *p*-value < 0.01 § REG1B, regenerating 1B protein.

Table S4. Associations between breastfeeding, antibiotic use, and dietary intervention and intestinal biomarkers in multivariable analysis †.

Variable	Calprotectin		§ REG1B		Alpha-1-antitrypsin	
	Coef. (95%CI)	*p*-Value	Coef. (95%CI)	*p*-Value	Coef. (95%CI)	*p*-Value
Continued breastfeeding (vs. no breastfeeding)	−0.19(−0.58,0.21)	0.350	0.49(−0.09,1.08)	0.098	−0.28(−0.59,0.03)	0.071
Antibiotic use (above vs. below median days)	−0.31(−0.50,−0.11)	0.002**	−0.01(−0.31,0.30)	0.964	−0.21(−0.36,−0.05)	0.009**
Dietary intervention (SQ-LNS vs. no supplements)	−0.04(−0.23,0.15)	0.685	0.06(−0.24,0.37)	0.689	−0.00(−0.16,0.16)	0.979

† Results were from multivariable regression models. ***p*-value < 0.01. § REG1B, regenerating 1B protein.
